# Altered Reward Circuit Function Moderates the Relationship between Childhood Maltreatment and Depression Severity in Adolescents

**DOI:** 10.1155/2023/4084004

**Published:** 2023-09-21

**Authors:** Weicheng Li, Zhibo Hu, Chengyu Wang, Xiaofeng Lan, Ming Zhang, Fan Zhang, Zerui You, Yanxiang Ye, Haiyan Liu, Zhanjie Luo, Yexian Zeng, Yiying Chen, Yifang Chen, Kai Wu, Guohui Lao, Jun Chen, Guixiang Li, Yanling Zhou, Yuping Ning

**Affiliations:** ^1^Department of Child and Adolescent Psychiatry, Affiliated Brain Hospital of Guangzhou Medical University, Guangzhou, China; ^2^The First School of Clinical Medicine, Southern Medical University, Guangzhou, China; ^3^Guangdong Engineering Technology Research Center for Translational Medicine of Mental Disorders, Guangzhou, China; ^4^Key Laboratory of Neurogenetics and Channelopathies of Guangdong Province and the Ministry of Education of China, The Second Affiliated Hospital, Guangzhou Medical University, Guangzhou, China; ^5^School of Biomedical Sciences and Engineering, South China University of Technology, Guangzhou, China; ^6^Guangdong Institute of Medical Instruments, Guangzhou, China; ^7^Institute of Biological and Medical Engineering, Guangdong Academy of Sciences, Guangzhou, China

## Abstract

**Background:**

The role of childhood maltreatment (CM) is believed to be crucial in the aberrant function of reward circuit in adolescent-onset major depressive disorder (AO-MDD). Nevertheless, the impact of abnormalities in the GP-based reward circuit on the association between CM and the severity of AO-MDD remains largely unknown.

**Methods:**

The GP-based resting-state functional connectivity (RSFC) was analyzed in a sample of 75 patients with AO-MDD and 80 healthy controls in order to identify potential abnormalities in the GP-based reward circuit in AO-MDD patients. Furthermore, we investigated the possible associations between aberrant GP-based reward circuit functioning, CM and its subtypes (namely, childhood abuse and childhood neglect), and the severity of AO-MDD.

**Results:**

Compared to the healthy control group, patients with AO-MDD demonstrated a reduction in RSFC between the left posterior GP and the right dorsomedial prefrontal cortex (DMPFC). Our moderation analysis revealed that the abnormal RSFC between the posterior GP and DMPFC had a moderating effect on the relationship between CM and the severity of AO-MDD. Furthermore, upon further interaction decomposition, we observed a positive correlation between CM and AO-MDD severity exclusively in patients with AO-MDD who exhibited lower RSFC between the posterior GP and DMPFC. For AO-MDD patients with higher RSFC between posterior GP and DMPFC, the relationship between CM and AO-MDD severity was not discernible.

**Conclusions:**

Our findings underscore the crucial role of anomalies in the reward circuit in AO-MDD and furnish novel leads for probing the relationship among CM, malfunctioning of the reward circuit, and AO-MDD.

## 1. Introduction

In the past few years, there has been a significant surge in the incidence of adolescent-onset major depressive disorder (AO-MDD), with a 12-month depression prevalence of about 13% [1]. Adolescence is a crucial stage of quick emotional and cognitive growth, and AO-MDD can significantly impede neurodevelopment and social functioning (such as academic progress and interpersonal relationships) [2]. Given its strong correlation with dropping out of school, nonsuicidal self-harm, persistent ailment, and recurrent depressive episodes in adulthood, AO-MDD has emerged as a growing societal issue [3, 4].

Childhood maltreatment (CM), which includes emotional, physical, and sexual abuse, as well as emotional and physical neglect, is a prevalent and potent risk element in the development of AO-MDD. Previous research has demonstrated that CM impacts over 35% of the worldwide population [5] and 45.6% of depressed patients have a history of CM [6]. AO-MDD patients exposed to CM often display an earlier onset of symptoms, more cooccurring disorders, and an inadequate response to clinical treatment [6, 7]. However, meta-analytic evidence suggests that traumatic childhood experiences are preventable and that AO-MDD patients could benefit from active treatment [8]. Consequently, as a disruptive yet positively modifiable aspect, CM should be accorded high priority in the intervention strategies of AO-MDD [8].

Clinical investigations have unveiled variegated responses to traumatic experiences among patients with AO-MDD [9, 10], indicating the existence of a subgroup of AO-MDD patients who are highly susceptible to the adverse effects of traumatic experiences, i.e., highly sensitive to CM. The neurobiological mechanisms that underlie hypersensitivity to CM in AO-MDD remain unclear, but certain clues suggest that it may be associated with aberrant resting-state functional connectivity (RSFC) of the reward circuit [11, 12]. The reward circuit is involved in the processing of rewarding effects (e.g., pleasure and euphoria), and the upregulation of dopamine system function in the reward circuit increases euphoric activity [13]. The core of the reward circuit is comprised of the globus pallidus (GP) and the nucleus accumbens, which receive signal input from the prefrontal cortex, hippocampus, and amygdala [14, 15]. While measuring the electrophysiological activity of the GP and nucleus accumbens in rats performing reward-based tasks, Ottenheimer et al. observed a greater number of reward-sensitive neurons within the GP compared to those in the nucleus accumbens [16]. Furthermore, a preference-related signal that flexibly reports the relative value of reward outcomes across multiple conditions was discovered in the GP [16]. These findings underscore the pivotal role of the GP in the reward circuit, suggesting that the GP is not simply a relay station for downstream signals from the nucleus accumbens but rather a core brain region within the reward circuit [16].

Previous research has demonstrated the adverse effects of CM on the reward circuit, specifically in the GP region. For example, Dillon et al. have shown that individuals who have been maltreated exhibit increased anhedonia and decreased responsivity of the GP towards rewarding stimuli [17]. However, it is important to note that the GP is not a homogenous structure, rather it is comprised of functionally and structurally heterogeneous nuclei. In a recent study, Tian et al. have constructed a novel set of brain atlases that apply to subcortical nuclei, based on a sample of over 1,000 healthy adults [18]. This pioneering work deviates from previous approaches that relied on structural magnetic resonance imaging (MRI) to map brain anatomy, as it utilizes functional MRI to elucidate the intricate topographical organization of the human subcortex, culminating in one of the most detailed gradient atlases of the subcortex to date [18]. According to the brain atlases derived by Tian et al., the GP is segmented into four subregions, which are the bilateral anterior globus pallidus (aGP) and the bilateral posterior globus pallidus (pGP) [18]. The aGP primarily serves motivational and cognitive functions [19], while the pGP is mainly involved in regulating motor functions, particularly the precision grip movement parameters [20].

The RSFC analysis represents a fundamental method for characterizing interregional brain activity, as it allows the examination of temporal correlations of blood oxygen level-dependent signals among different brain regions [21]. RSFC analysis provides more objective evidence, shedding light on significant pathophysiological processes in depression [22]. In recent years, RSFC has been employed to investigate functional abnormalities in the brains of individuals with MDD with CM [23, 24]. In the present study, we selected four subregions of the GP in the bilateral hemispheres as regions of interest (ROI) for RSFC analysis, with the aim of exploring abnormalities in the reward circuit of patients with AO-MDD. Specifically, we aimed to investigate the relationship between CM, altered function of the reward circuit, and the outcomes of AO-MDD. We generated the following hypothesis: (i) compared to healthy adolescents, patients with AO-MDD exhibit anomalous reward circuit function that is centered on the GP, and (ii) in patients with AO-MDD, altered reward circuit function may play a critical role in explaining the relationship between CM and the severity of depression. Our study contributes new evidence to confirm the presence of abnormal reward circuit in AO-MDD and provides additional neuroimaging clues that may aid in identifying AO-MDD patients who are highly sensitive to traumatic experiences.

## 2. Methods

### 2.1. Participants

Seventy-five patients diagnosed with AO-MDD and 80 healthy controls (HCs) were included in the baseline data of a registered clinical trial (ChiCTR2100042346). The study received approval from the Ethics Committee of the Affiliated Brain Hospital of Guangzhou Medical University, and all participants and their parents provided written informed consent. Both AO-MDD patients and HCs underwent a structured clinical interview based on the diagnostic and statistical manual of mental disorders, fifth edition (DSM-5), and the diagnosis of major depressive disorder was made by a trained clinician. All patients met the following inclusion criteria: (i) age between 12 and 18 years old, (ii) first episode of AO-MDD, (iii) 17-item Hamilton Rating Scale for Depression (HAMD-17) score ≥ 17, and (iv) had been medication-free for at least 4 weeks prior to inclusion in the trial. First episode and unmedicated AO-MDD patients were recruited from the outpatient department of the Affiliated Brain Hospital of Guangzhou Medical University. Healthy controls matched for age and education level were recruited through advertising. Participants with a history of developmental disorders, tic disorders, or attention deficit hyperactivity disorder were excluded, as were those with any other mental illness (e.g., bipolar disorder, posttraumatic stress disorder, and schizophrenia), neurological disorders or other major physical illnesses, and contraindications to MRI scanning.

The Hamilton Rating Scale for Depression (HAMD-17) was employed to evaluate the severity of depression in AO-MDD [25]. Meanwhile, the Childhood Trauma Questionnaire (CTQ) was utilized for measuring CM, encompassing two major categories of traumatic experiences, namely, childhood neglect and childhood abuse [26, 27]. The average score of all 25 items of the CTQ served as the comprehensive CM score (range = 1.16 ~ 3.36). The score for childhood abuse was determined by the average score of 15 items that assessed abuse (range = 1.00 ~ 3.33), while the score for childhood neglect was calculated based on the average score of 10 items that evaluated neglect (range = 1.30 ~ 4.20).

### 2.2. Definition of Regions of Interest

In the seed-based RSFC analysis, four subregions of the GP, comprising the bilateral aGP and bilateral pGP, were designated as the ROI based on the brain atlas mapped by Tian et al. The anatomical position of the GP and its subregions are delineated in Figure S1.

### 2.3. MRI Data Acquisition

All images were acquired using a Siemens Magnetom Prisma 3.0T MRI Scanner, equipped with a 64-channel head coil, at the Magnetic Resonance Center of Affiliated Brain Hospital of Guangzhou Medical University. Participants were provided with instructions to maintain eye closure and wakefulness and minimize cognitive activity during the scanning procedure. The whole-brain functional echo-planar images were obtained transversely with a gradient-recalled echo-planar imaging pulse sequence utilizing the following parameters: repetition time = 800 ms, echo time = 30 ms, flip angle =56°, slice thickness = 2 mm, number of slices = 72, and field of view = 208 × 208 mm. The anatomical image was obtained utilizing a sagittal three-dimensional gradient echo T1-weighted sequence with the following settings: repetition time = 2000 ms, echo time = 2.32 ms, inversion times = 900 ms, flip angle =8°, number of slices = 208, slice thickness = 0.9 mm, and matrix = 256 × 256.

### 2.4. MRI Data Preprocessing

The functional images underwent preprocessing using Data Processing and Analysis for Brain Imaging (DPABI_V6.1, http://rfmri.org/dpabi), which was implemented in MATLAB (version R2019b). Signal stabilization was achieved by removing the first 25 functional volumes, and the remaining 425 volumes were corrected for timing differences between slices. Motion correction of the functional images was accomplished using a six motion parameter (rigid body). It is noteworthy that the mean framewise displacement (FD) based on the Jenkinson model (FD-Jenkinson) was computed by averaging the FD from every time point for each participant [28]. No participants with head movement exceeding 2 mm or rotation exceeding 2° were excluded. Following motion correction, the individual structural image (T1-weighted image) was coregistered to the mean functional image. The transformed structural images were then segmented into gray matter, white matter, and cerebrospinal fluid. The rigid-body six model, the white matter signal, and the cerebrospinal fluid signal were subsequently treated as nuisance covariates and regressed out. Finally, the images were temporally band-pass filtered between 0.01 Hz and 0.08 Hz and smoothed using a 4 mm full width at half maximum Gaussian kernel.

### 2.5. Calculation of RSFC with Globus Pallidus as Regions of Interest

The RSFC analysis was executed utilizing the DPABI software. The RSFC analysis with the four subregions of the GP as ROI comprised of a three-step approach. Firstly, the time series within the selected ROIs were extracted. Subsequently, a voxel-wise correlation analysis was carried out between the subregions of GP and other brain areas to derive RSFC maps. Finally, the obtained RSFC maps underwent a Z-transformation to enhance the normality of the data distribution.

### 2.6. Statistical Analysis

In this study, the statistical analysis can be segregated into four distinct components as outlined below.

- Differences in demographic characteristics between groups, such as age, gender, education level, and duration of the disease, as well as clinical characteristics such as HAMD-17 score and CTQ score, were analyzed using *t*-tests and *χ*^2^ tests with SPSS 23.0 (IBM Corporation, Armonk, NY, USA)
- We utilized two-sample *t*-tests to examine differences between patients with AO-MDD and HCs in RSFC analysis with the GP serving as the ROI. To control for potential confounding variables, we included gender, age, education level, and mean FD as covariates. Additionally, given that the majority of participants were female (59%), we conducted a validation analysis for females to verify the robustness of our results (refer to supplementary materials). Gaussian random field (GRF) correction was performed with a significance threshold of voxel-*p* < 0.001 and cluster-*p* < 0.05. Additional analysis was conducted to investigate the relationship between altered reward circuit function and the severity of anhedonia, as measured by the score of item 7 of the HAMD-17. In specificity analyses, we explored whether analogous results would emerge for the accumbens (comprising the nucleus accumbens shell and nucleus accumbens core)
- Correlational analyses were performed separately on AO-MDD patients and HCs to investigate the relationship between the severity of depression (including dimensions such as depression severity, anxiety/somatization, retardation, cognitive disturbance, sleep disruption, and weight) and traumatic experiences (such as the CTQ total score, childhood abuse score, and childhood neglect score)
- Multiple linear regression was further employed to investigate whether the abnormal RSFC between dorsomedial prefrontal cortex (DMPFC) and pGP (serving as moderating variable *M*) moderated the relationship between traumatic experiences (serving as independent variable *X*, encompassing childhood abuse, childhood neglect, and CTQ total score) and AO-MDD severity (serving as dependent variable *Y*) in AO-MDD patients and HCs. Covariates such as age, education, and gender were treated as controlling variables. Traumatic experiences and abnormal RSFC between DMPFC and pGP were considered continuous variables in all models. In the post hoc specificity analysis, anxiety severity (represented by HAMA score) was utilized as an alternative outcome variable, with traumatic experiences serving as the independent variable and abnormal RSFC between DMPFC and pGP as the moderating variable

## 3. Results

### 3.1. Demographic and Clinical Characteristics

The demographic and clinical characteristics of all participants are delineated in Table 1. Notably, AO-MDD patients did not exhibit any significant difference from HCs in relation to age, education level, and mean FD. However, there was a significant difference between the two groups with regard to gender, which warranted consideration as a covariate to mitigate potential confounding effects. Furthermore, significant differences were observed between AO-MDD patients and HCs in relation to HAMD-17 total score, HAMA, CTQ total score, childhood abuse score, and childhood neglect score.

### 3.2. Correlation Analysis between Depression Severity and CM

Correlation analyses were conducted to investigate the relationship between depression severity and CM in AO-MDD patients and HCs. Specifically, the HAMD-17 total score and its dimensions (namely, depression severity, anxiety/somatization, retardation, cognitive disturbance, sleep disruption, and weight) were analyzed alongside the CTQ total score and its subscales (including childhood abuse and childhood neglect). As demonstrated in Figure S2, the HAMD-17 total score exhibited a significant association with childhood abuse, while sleep disruption was significantly associated with the CTQ total score in AO-MDD patients. However, in HCs, we only observed a correlation between the HAMD-17 total score and childhood abuse.

### 3.3. Abnormal RSFC with Globus Pallidus as Regions of Interest in AO-MDD Patient

**Figure 1:**
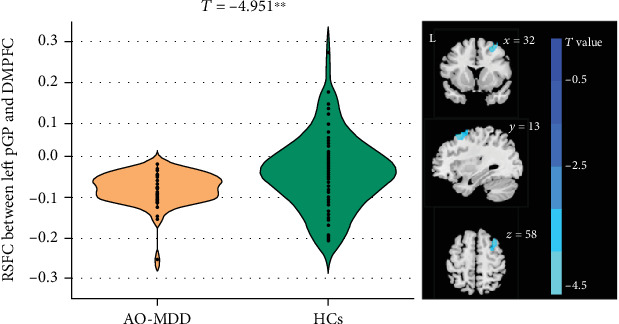
Between-group differences in RSFC of left posterior globus pallidus and right DMPFC between AO-MDD and HCs. Abbreviations: AO-MDD: adolescent-onset major depressive disorder; HCs: heathy controls; RSFC: resting-state functional connectivity; pGP: posterior globus pallidus; DMPFC: dorsolateral prefrontal.

Analyses of two-sample *t*-test were conducted to examine variations in ROI-based RSFC between patients with AO-MDD and HCs. As illustrated in Figure 1 and Table S1, there was a statistically significant decrease in RSFC between the left pGP and the DMPFC (BA8) in AO-MDD patients compared to HCs. However, there was no significant difference between AO-MDD patients and HCs in the RSFC analyses involving the bilateral aGP and the right pGP as ROIs. We examined the RSFC of DMPFC-pGP in relation to anhedonia severity, which was assessed using the score of item 7 of the HAMD-17. Our analysis revealed a close-to-significant association with anhedonia severity, with an *r*-value of 0.199 and a *p* value of 0.086. Additionally, we did not observe any significant differences between AO-MDD patients and HCs in the RSFC analyses involving the four subregions of the nucleus accumbens as ROIs.

### 3.4. Moderation Analysis

Table 2 presents the results of the multiple linear regression analysis conducted on AO-MDD patients to investigate the impact of traumatic experiences on AO-MDD severity. Model 1 highlights that the interaction between RSFC of DMPFC-pGP and CTQ total score had a significant effect on AO-MDD severity. In model 2, the interaction between RSFC of DMPFC-pGP and childhood abuse was also found to be significant. However, the interaction between RSFC of DMPFC-pGP and childhood neglect in model 3 did not reach statistical significance. Additionally, Table S2 of the supplementary material shows that no significant interaction effects were detected in the HCs. Therefore, the results presented below pertain exclusively to the dataset of AO-MDD patients.

**Figure 2:**
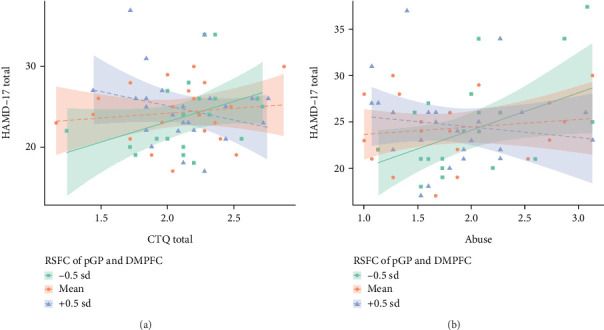
RSFC of left pGP and DMPFC as moderators of the relationship between traumatic experiences (including total CTQ score/childhood abuse) and AO-MDD severity. Separate lines for -0.5, 0, and +0.5 SD from the sample RSFC mean are for visualization purposes only. Abbreviations: HAMD-17: 17-item Hamilton Depression Rating Scale; CTQ: Childhood Trauma Questionnaire; RSFC: resting-state functional connectivity; pGP: posterior globus pallidus; DMPFC: dorsolateral prefrontal.

To further decompose the interaction effects of RSFC between the DMPFC and the pGP, as well as traumatic experiences (given that model 3 failed to attain significance, only the CTQ total score and childhood abuse were incorporated) on the severity of AO-MDD, patients with AO-MDD were stratified into high, medium, and low reward circuit function groups. Additional multiple linear regressions were executed individually in each subgroup, wherein high, medium, and low reward circuit functions were denoted by high, medium, and low RSFC between DMPFC and pGP, respectively. Specifically, the low reward circuit function group encompassed AO-MDD patients exhibiting RSFC between DMPFC and pGP < −0.0424 (below mean -0.5 standard deviation (SD)), the medium reward circuit function group comprised AO-MDD patients displaying RSFC between DMPFC and pGP ≤ −0.0424 and < -0.0040 (mean -0.5 SD to mean +0.5 SD), and the high reward circuit function group included AO-MDD patients possessing RSFC between DMPFC and pGP ≥ −0.0040 (above mean +0.5 SD). The mean ±0.5 SD threshold was selected to ensure similarity of participant numbers in each subgroup. These multiple linear regressions treated CTQ total score and childhood abuse as independent variables, AO-MDD severity as the dependent variable, and gender, age, and education level as covariates. As evinced in Tables 3 and 4 and Figure 2, CTQ total score and childhood abuse manifested a significant association with AO-MDD severity solely in the low reward circuit function group, and the more severe the traumatic experiences, the more severe the AO-MDD.

To investigate whether the moderating impact of aberrant RSFC between the DMPFC and pGP was exclusive to the severity of AO-MDD, we conducted an additional moderation analysis. In this analysis, we utilized anxiety severity, as determined by HAMA scores, as the dependent variable, traumatic experience as the independent variable, and aberrant RSFC between the DMPFC and pGP as the moderating variable. Subsequently, a post hoc specificity analysis revealed that the moderating impact of aberrant RSFC between the DMPFC and pGP was only observed with respect to the severity of AO-MDD and not in relation to anxiety severity. This indicates that the moderating effect of abnormal RSFC between the DMPFC and pGP is specific to the severity of AO-MDD (refer to Table S3 for details).

### 3.5. Validation

Furthermore, given that a majority of our study participants were of the female sex (59%), we conducted the aforementioned analyses solely on female subjects to ascertain the robustness of our results. As depicted in Table S3 of the supplementary materials, we observed that our findings remained unaffected by any potential gender bias.

## 4. Discussion

In the present study, we examined the aberrant RSFC with the GP as the ROI in patients with AO-MDD. Our aim was to explore the relationship between CM, AO-MDD severity, and the abnormal functioning of the reward circuit centered on the GP. The following findings were obtained: (i) A decrease in the RSFC between the pGP and the DMPFC was observed in AO-MDD patients compared to HCs, suggesting that patients with AO-MDD had an aberrant reward circuit function with the GP as its core. (ii) The association between CM and AO-MDD severity was moderated by the abnormal functioning of the reward circuit centered on the GP. In AO-MDD patients with reduced reward circuit function, greater CM was associated with increased severity of AO-MDD. (iii) Similarly, reward circuit abnormalities moderated the association between childhood abuse and AO-MDD severity. However, this moderating effect of reward circuit abnormalities was not observed between childhood neglect and AO-MDD severity. (iv) Post hoc specificity analysis revealed that the moderating effect of reward circuit abnormalities was observed only between CM/childhood abuse and AO-MDD severity, but not between CM and anxiety severity. This indicated that the moderating effect of reward circuit abnormalities was specific to depression severity in AO-MDD patients. Furthermore, validation analysis demonstrated that our findings were not influenced by gender bias, indicating that our results were robust. Our examination of reward circuits in individuals with AO-MDD enhances the understanding of the diminished positive emotions observed in AO-MDD and offers novel insights into the neural mechanisms underlying mood and subjective experience in this population.

Most prior investigations concerning reward anticipation have focused on abnormalities in the reward circuit associated with the nucleus accumbens, while disregarding the significant role of the GP in this circuit. Building on Ottenheimer et al.'s innovative proposal for a reward circuit centered on the GP [16], the present study provides the first confirmation that patients with AO-MDD demonstrate reduced RSFC between the pGP and DMPFC, offering novel evidence of abnormal reward circuit in AO-MDD patients. There is prior research that has shown that within the reward circuitry, information flow from the ventral striatum (where the GP is a component) can project to the frontal cortex, including the prefrontal regions [29]. The pGP is recognized as a critical region for motor and motivational functions, as it receives inputs from various reward-related neural circuits, effectively linking goal-driven behaviors with the anticipation of rewards [30]. Animal models of adverse life events have revealed alterations in reward-seeking behaviors [31] and increased self-administration of various psychoactive substances [32]. In addition, Pechtel and Pizzagalli found that females who had experienced childhood sexual abuse exhibited lower behavioral accuracy when utilizing learned information in reward tasks [33]. Additionally, we observed that reduced reward circuit function moderated the relationship between CM and AO-MDD severity, lending support to the notion that not all AO-MDD patients who experience CM suffer significant negative effects from traumatic experiences and that AO-MDD patients with diminished reward circuit function are more sensitive to CM. These findings are consistent with prior research; for example, a prospective cohort study by Vidal-Ribas et al. found that exposure to stressful life events during childhood predicted reduced neural responses to reward anticipation in basal ganglia regions, particularly the nucleus accumbens and GP [34]. Additionally, diminished responses to reward anticipation in the medial prefrontal and anterior cingulate cortex predicted greater stress reactivity in the future [34]. This offers evidence for a bidirectional association between stress and reward circuit function, whereby early stress may impact reward anticipation, while reduced reward anticipation may increase susceptibility to stress [34]. Furthermore, Ding et al. discovered that recurrent MDD patients exhibit reduced nucleus accumbens RSFC in both the reward network and default mode network [35]. Taken together with prior research, our findings confirm that dysregulation of corticosubcortical connectivity may be a plausible mechanism for abnormal reward processing in AO-MDD [36].

We have observed that abnormalities in the reward circuit moderated the association between childhood abuse and the severity of AO-MDD. Specifically, we found that the impact of childhood abuse on AO-MDD severity was attenuated in patients exhibiting higher reward circuit function. Conversely, we did not identify a moderating effect of reward circuit abnormalities on the relationship between childhood neglect and AO-MDD severity, indicating that childhood abuse and childhood neglect have differential effects on patients with AO-MDD. Prior investigations have reported similar findings, namely, that childhood abuse is strongly associated with affective symptoms, while childhood neglect is highly correlated with cognitive symptoms [37, 38]. For example, Kilian et al. have demonstrated that a history of childhood neglect is a significant predictor of impaired social cognition in adulthood, including attention/agility and verbal learning ability [37]. Furthermore, children who experience childhood abuse, particularly physical and sexual abuse, are more susceptible to developing symptoms of emotional dysregulation in adulthood, such as impulsive aggression, repeated self-harm, and chronic suicidal tendencies [39]. An important and as-yet-unanswered question is why childhood abuse and neglect have differing effects on AO-MDD patients. Existing research suggests that the neurobiological mechanisms underlying the effects of childhood abuse and neglect on AO-MDD patients are distinct [40, 41], with childhood abuse exerting a more significant impact on reward circuit function in patients with AO-MDD [42]. Our discovery that abnormal reward circuits modulate the relationship between childhood abuse and AO-MDD severity provides novel insights into exploring the underlying neural mechanisms in AO-MDD patients who have suffered childhood abuse.

Upon performing a post hoc specificity analysis, it was discovered that the moderating effect of abnormalities within the reward circuit was exclusively evident in the association between CM and the severity of AO-MDD, while no such effect was observed between CM and anxiety severity. This suggests that the moderating effect of reward circuit abnormalities is specific to the severity of AO-MDD, which may be due to the distinct neurobiological mechanisms underlying depressive and anxiety symptoms. Using a mouse model, Fan et al. discovered a strong link between depressive-like states associated with social status loss and abnormal function of the reward circuit [43]. Soares-Cunha et al. found that subtypes of spiny neurons located in the nucleus accumbens, a critical area of the reward circuit, signal both reward and aversion to regulate depression-like symptoms [44]. Additionally, Morel et al. utilized the chronic social defeat stress paradigm to reveal that the midbrain projection to the basolateral amygdala encodes anxiety-like behaviors, but not depression-like behaviors [45]. In conjunction with prior research, we suggest that the decreased function of the reward circuit is related to the underlying neurobiological mechanisms of depression severity, while overactivation of the amygdala-related pathway is associated with anxiety symptoms.

The present study is subject to certain limitations. Firstly, our study's cross-sectional design does not permit us to draw inferences regarding the causal relationships among variables. Secondly, due to the limited sample size, we were unable to categorize patients with AO-MDD into AO-MDD with and without CM groups based on their exposure to traumatic events. Thirdly, the evaluation of CM is based on retrospective questionnaires, which may be subject to personal bias and memory distortion. However, it is pertinent to note that self-reported accounts of CM are considered valid in the absence of objective evidence, as they represent an individual's perception of early-life adversity [46]. Fourthly, due to the gender mismatch between the AO-MDD patients and the HCs, gender was accounted for as a covariate. To assess whether gender bias influenced our findings, we performed a validation analysis exclusively on female participants. Our results, as demonstrated in the Supplementary Material, were not impacted by gender bias.

In sum, we have observed a decline in RSFC between the left pGP and the DMPFC among patients with AO-MDD. This suggests that patients with AO-MDD have a weakened reward circuit function centered on the GP. Furthermore, the analysis of moderation and interaction decomposition indicated that AO-MDD patients with lower reward circuit function exhibited higher susceptibility to CM, i.e., hypersensitivity to traumatic experiences. Our findings emphasize the crucial role of reward circuit abnormalities in the onset of AO-MDD and offer new leads to explore the relationship between CM, reward circuit dysfunction, and AO-MDD.

## Figures and Tables

**Table 1:** Demographic and clinical features of participants.

Variables	AO-MDD (*n* = 75)	HCs (*n* = 80)	*T*/*χ*^2^	*p*
Gender (male/female)	61/14	31/49	29.096	<0.001
Age (years)	14.76 ± 2.09	14.90 ± 1.97	-0.429	0.669
Education (years)	8.88 ± 2.01	9.01 ± 2.01	-0.410	0.682
Duration of illness (month)	18.64 ± 14.87	—	—	—
HAMD-17	24.35 ± 4.43	1.33 ± 2.28	40.242	<0.001
HAMA	26.28 ± 6.55	0.88 ± 1.28	33.02	<0.001
CTQ total	2.14 ± 0.39	1.13 ± 0.29	14.240	<0.001
Abuse	1.89 ± 0.60	1.12 ± 0.18	10.253	<0.001
Neglect	2.52 ± 0.63	1.65 ± 0.60	8.633	<0.001
Mean framewise displacement	0.07 ± 0.03	0.07 ± 0.03	0.616	0.539

Abbreviations: AO-MDD: adolescent-onset major depressive disorder; HCs: heathy controls; HAMD-17: 17-item Hamilton Depression Rating Scale; CTQ: Childhood Trauma Questionnaire, Hamilton Anxiety Scale.

**Table 2:** Multiple linear regression of HAMD-17 total by CTQ total and abuse and neglect with RSFC as a moderator.

	Estimate	SE	*T*	*p*
Model 1: CTQ total				
Intercept	33.3208	6.6458	5.0138	<0.0001
Age	-0.0571	0.5117	-0.1116	0.9115
Education	-0.2300	0.5358	-0.4293	0.6692
Female sex	-2.1604	1.3715	-1.5752	0.1203
CTQ	-0.8725	1.8696	-0.4667	0.6424
RSFC	188.2752	84.5123	2.2278	0.0295
CTQ total × RSFC	-84.6361	37.5215	-2.2557	0.0276

Model 2:abuse				
Intercept	30.7182	5.3559	5.7354	<0.0001
Age	0.0181	0.4875	0.0371	0.9706
Education	-0.2346	0.5130	-0.4573	0.6491
Female sex	-2.1657	1.3275	-1.6314	0.1079
Abuse	-0.1671	1.0686	-0.1564	0.8762
RSFC	163.0415	57.9125	2.8153	0.0065
Abuse × RSFC	-79.3071	27.6931	-2.8638	0.0057

Model 3:neglect				
Intercept	33.2946	6.3723	5.2249	<0.0001
Age	0.1452	0.5395	0.2692	0.7887
Education	-0.4737	0.5585	-0.8481	0.3996
Female sex	-2.1326	1.4392	-1.4818	0.1435
Neglect	-1.1025	1.1927	-0.9243	0.3589
RSFC	72.8072	75.0155	0.9706	0.3355
Neglect × RSFC	-29.1675	29.1177	-1.0017	0.3204

Abbreviations: HAMD-17: 17-item Hamilton Depression Rating Scale; RSFC: resting-state functional connectivity.

**Table 3:** Multiple linear regression of HAMD-17 total by CTQ total by RSFC group.

	Estimate	SE	*T*	*p*
Model 1: RSFC < −0.0424 (*n* = 22)				
Intercept	6.3960	13.8080	0.4630	0.6490
Age	-0.4060	0.8840	-0.4600	0.6510
Education	0.9000	0.9500	0.9470	0.3570
Female sex	1.2790	3.1790	0.4020	0.6930
CTQ total	6.0710	2.3740	2.5580	0.0200

Model 2: −0.0424 < RSFC < −0.0040 (*n* = 25)				
Intercept	14.2990	10.6860	1.3380	0.1980
Age	1.0380	1.4110	0.7360	0.4720
Education	-0.8280	1.6670	-0.4970	0.6260
Female sex	-1.4810	2.5530	-0.5800	0.5700
CTQ total	2.2100	2.8430	0.7770	0.4480

Model 3: RSFC > −0.0040 (*n* = 22)				
Intercept	63.8410	13.7510	4.6420	<0.0001
Age	-1.9090	1.1930	-1.6000	0.1250
Education	1.2570	1.2220	1.0290	0.3160
Female sex	-8.4400	3.6320	-2.3240	0.0310
CTQ total	-2.6680	2.5040	-1.0660	0.2990

Abbreviations: HAMD-17: 17-item Hamilton Depression Rating Scale; RSFC: resting-state functional connectivity; CTQ: Childhood Trauma Questionnaire.

**Table 4:** Multiple linear regression of HAMD-17 total by abuse by RSFC group.

	Estimate	SE	*T*	*p*
Model 1: RSFC < −0.0424 (*n* = 22)				
Intercept	6.9665	13.4032	0.5200	0.6100
Age	0.0855	0.8636	0.0990	0.9220
Education	0.5060	0.9051	0.5590	0.5830
Female sex	1.1468	3.1268	0.3670	0.7180
Abuse	4.7433	1.7494	2.7110	0.0150

Model 2: −0.0424 < RSFC < −0.0040 (*n* = 25)				
Intercept	12.7274	9.4632	1.3449	0.1963
Age	0.8520	1.2318	0.6917	0.4985
Education	-0.3225	1.5065	-0.2141	0.8330
Female sex	-1.5383	2.1929	-0.7015	0.4925
Abuse	2.5342	1.5437	1.6416	0.1190

Model 3: RSFC > −0.0040 (*n* = 22)				
Intercept	58.9905	13.2899	4.4387	0.0003
Age	-1.7826	1.2373	-1.4407	0.1652
Education	1.1226	1.2626	0.8892	0.3845
Female sex	-8.8242	3.7389	-2.3601	0.0285
Abuse	-0.4184	1.5376	-0.2721	0.7883

Abbreviations: HAMD-17: 17-item Hamilton Depression Rating Scale; RSFC: resting-state functional connectivity.

## Data Availability

The data that support the findings of this study are available from the corresponding authors upon reasonable request.

## Supplementary Materials

Supplementary Materials Figure S1: visualization of the subregions of globus pallidus. Four seeds of the globus pallidus in the bilateral hemisphere. Figure S2: correlation analysis between depression severity and CM in the AO-MDD and HC groups. Table S1: regions showing significant differences in functional connectivity among the AO-MDD and HC groups (*n* = 155). Table S2: multiple linear regression of HAMA total by CTQ total and abuse and neglect with RSFC as a moderator. Table S3: regions showing significant differences in functional connectivity among the AO-MDD and HC groups in female participants (*n* = 92).

## References

[1] Kalin N. H. (2021). Anxiety, depression, and suicide in youth. *The American Journal of Psychiatry*.

[2] Davey C. G., McGorry P. D. (2019). Early intervention for depression in young people: a blind spot in mental health care. *Lancet Psychiatry*.

[3] Liu R. T., Walsh R., Sheehan A. E., Cheek S. M., Sanzari C. M. (2022). Prevalence and correlates of suicide and nonsuicidal self-injury in children: a systematic review and meta-analysis. *JAMA Psychiatry*.

[4] Miller L., Campo J. V. (2021). Depression in adolescents. *The New England Journal of Medicine*.

[5] Hughes K., Bellis M. A., Hardcastle K. A. (2017). The effect of multiple adverse childhood experiences on health: a systematic review and meta-analysis. *The Lancet Public Health*.

[6] Nelson J., Klumparendt A., Doebler P., Ehring T. (2017). Childhood maltreatment and characteristics of adult depression: meta-analysis. *The British Journal of Psychiatry*.

[7] Nanni V., Uher R., Danese A. (2012). Childhood maltreatment predicts unfavorable course of illness and treatment outcome in depression: a meta-analysis. *The American Journal of Psychiatry*.

[8] Kuzminskaite E., Gathier A. W., Cuijpers P. (2022). Treatment efficacy and effectiveness in adults with major depressive disorder and childhood trauma history: a systematic review and meta-analysis. *The Lancet Psychiatry*.

[9] Herringa R. J., Birn R. M., Ruttle P. L. (2013). Childhood maltreatment is associated with altered fear circuitry and increased internalizing symptoms by late adolescence. *Proceedings of the National Academy of Sciences of the United States of America*.

[10] Redlich R., Opel N., Burger C. (2018). The limbic system in youth depression: brain structural and functional alterations in adolescent in-patients with severe depression. *Neuropsychopharmacology*.

[11] Guyer A. E., Kaufman J., Hodgdon H. B. (2006). Behavioral alterations in reward system function: the role of childhood maltreatment and psychopathology. *Journal of the American Academy of Child and Adolescent Psychiatry*.

[12] McCrory E. J., Gerin M. I., Viding E. (2017). Annual research review: childhood maltreatment, latent vulnerability and the shift to preventative psychiatry - the contribution of functional brain imaging. *Journal of Child Psychology and Psychiatry*.

[13] Su Y. A., Si T. (2022). Progress and challenges in research of the mechanisms of anhedonia in major depressive disorder. *General Psychiatry*.

[14] de Olmos J. S., Heimer L. (1999). The concepts of the ventral striatopallidal system and extended amygdala. *Annals of the New York Academy of Sciences*.

[15] Groenewegen H. J., Wright C. I., Beijer A. V., Voorn P. (1999). Convergence and segregation of ventral striatal inputs and outputs. *Annals of the New York Academy of Sciences*.

[16] Ottenheimer D., Richard J. M., Janak P. H. (2018). Ventral pallidum encodes relative reward value earlier and more robustly than nucleus accumbens. *Nature Communications*.

[17] Dillon D. G., Holmes A. J., Birk J. L., Brooks N., Lyons-Ruth K., Pizzagalli D. A. (2009). Childhood adversity is associated with left basal ganglia dysfunction during reward anticipation in adulthood. *Biological Psychiatry*.

[18] Tian Y., Margulies D. S., Breakspear M., Zalesky A. (2020). Topographic organization of the human subcortex unveiled with functional connectivity gradients. *Nature Neuroscience*.

[19] Pessiglione M., Schmidt L., Draganski B. (2007). How the brain translates money into force: a neuroimaging study of subliminal motivation. *Science*.

[20] Prodoehl J., Corcos D. M., Vaillancourt D. E. (2009). Basal ganglia mechanisms underlying precision grip force control. *Neuroscience and Biobehavioral Reviews*.

[21] Biswal B., Yetkin F. Z., Haughton V. M., Hyde J. S. (1995). Functional connectivity in the motor cortex of resting human brain using echo-planar MRI. *Magnetic Resonance in Medicine*.

[22] Spellman T., Liston C. (2020). Toward circuit mechanisms of pathophysiology in depression. *The American Journal of Psychiatry*.

[23] Luo Q., Chen J., Li Y. (2022). Aberrant static and dynamic functional connectivity of amygdala subregions in patients with major depressive disorder and childhood maltreatment. *Neuroimage: Clinical*.

[24] Teicher M. H., Samson J. A., Anderson C. M., Ohashi K. (2016). The effects of childhood maltreatment on brain structure, function and connectivity. *Nature Reviews. Neuroscience*.

[25] Hamilton M. (1960). A rating scale for depression. *Journal of Neurology, Neurosurgery, and Psychiatry*.

[26] Bernstein D. P., Fink L., Handelsman L. (1994). Initial reliability and validity of a new retrospective measure of child abuse and neglect. *The American Journal of Psychiatry*.

[27] Bernstein D. P., Stein J. A., Newcomb M. D. (2003). Development and validation of a brief screening version of the childhood trauma questionnaire. *Child Abuse & Neglect*.

[28] Jenkinson M., Bannister P., Brady M., Smith S. (2002). Improved optimization for the robust and accurate linear registration and motion correction of brain images. *NeuroImage*.

[29] Haber S. N., Knutson B. (2010). The reward circuit: linking primate anatomy and human imaging. *Neuropsychopharmacology*.

[30] Saga Y., Hoshi E., Tremblay L. (2017). Roles of multiple globus pallidus territories of monkeys and humans in motivation, cognition and action: an anatomical, physiological and pathophysiological review. *Frontiers in Neuroanatomy*.

[31] Hanson J. L., Williams A. V., Bangasser D. A., Pena C. J. (2021). Impact of early life stress on reward circuit function and regulation. *Frontiers in Psychiatry*.

[32] Lewis C. R., Staudinger K., Scheck L., Olive M. F. (2013). The effects of maternal separation on adult methamphetamine self-administration, extinction, reinstatement, and MeCP2 immunoreactivity in the nucleus accumbens. *Frontiers in Psychiatry*.

[33] Pechtel P., Pizzagalli D. A. (2013). Disrupted reinforcement learning and maladaptive behavior in women with a history of childhood sexual abuse: a high-density event-related potential study. *JAMA Psychiatry*.

[34] Vidal-Ribas P., Benson B., Vitale A. D. (2019). Bidirectional associations between stress and reward processing in children and adolescents: a longitudinal neuroimaging study. *Biological Psychiatry: Cognitive Neuroscience and Neuroimaging*.

[35] Ding Y. D., Chen X., Chen Z. B. (2022). Reduced nucleus accumbens functional connectivity in reward network and default mode network in patients with recurrent major depressive disorder. *Translational Psychiatry*.

[36] Ng T. H., Alloy L. B., Smith D. V. (2019). Meta-analysis of reward processing in major depressive disorder reveals distinct abnormalities within the reward circuit. *Translational Psychiatry*.

[37] Kilian S., Asmal L., Chiliza B. (2018). Childhood adversity and cognitive function in schizophrenia spectrum disorders and healthy controls: evidence for an association between neglect and social cognition. *Psychological Medicine*.

[38] van Dam D. S., van Nierop M., Viechtbauer W. (2015). Childhood abuse and neglect in relation to the presence and persistence of psychotic and depressive symptomatology. *Psychological Medicine*.

[39] Lieb K., Zanarini M. C., Schmahl C., Linehan M. M., Bohus M. (2004). Borderline personality disorder. *Lancet*.

[40] Nagy S. A., Kurtos Z., Nemeth N. (2021). Childhood maltreatment results in altered deactivation of reward processing circuits in depressed patients: a functional magnetic resonance imaging study of a facial emotion recognition task. *Neurobiology of Stress*.

[41] Puetz V. B., Viding E., Gerin M. I. (2020). Investigating patterns of neural response associated with childhood abuse v. childhood neglect. *Psychological Medicine*.

[42] Cassiers L., Sabbe B., Schmaal L., Veltman D. J., Penninx B., Van Den Eede F. (2018). Structural and functional brain abnormalities associated with exposure to different childhood trauma subtypes: a systematic review of neuroimaging findings. *Frontiers in Psychiatry*.

[43] Fan Z., Chang J., Liang Y. (2023). Neural mechanism underlying depressive-like state associated with social status loss. *Cell*.

[44] Soares-Cunha C., de Vasconcelos N., Coimbra B. (2020). Nucleus accumbens medium spiny neurons subtypes signal both reward and aversion. *Molecular Psychiatry*.

[45] Morel C., Montgomery S. E., Li L. (2022). Midbrain projection to the basolateral amygdala encodes anxiety-like but not depression-like behaviors. *Nature Communications*.

[46] Danese A., Widom C. S. (2020). Objective and subjective experiences of child maltreatment and their relationships with psychopathology. *Nature Human Behaviour*.

