# Supplementary material for: Altered Reward Circuit Function Moderates the Relationship between Childhood Maltreatment and Depression Severity in Adolescents
Source: Depress Anxiety. 2023 Sep 21;2023:4084004. doi: 10.1155/2023/4084004 (PMC11921849; doi:10.1155/2023/4084004)
Supplement: Supplementary Materials — Figure S1: visualization of the subregions of globus pallidus. Four seeds of the globus pallidus in the bilateral hemisphere. Figure S2: correlation analysis between depression severity and CM in the AO-MDD and HC groups. Table S1: regions showing significant differences in functional connectivity among the AO-MDD and HC groups (n = 155). Table S2: multiple linear regression of HAMA total by CTQ total and abuse and neglect with RSFC as a moderator. Table S3: regions showing significant differences in functional connectivity among the AO-MDD and HC groups in female participants (n = 92). [file 4084004.f1.docx]

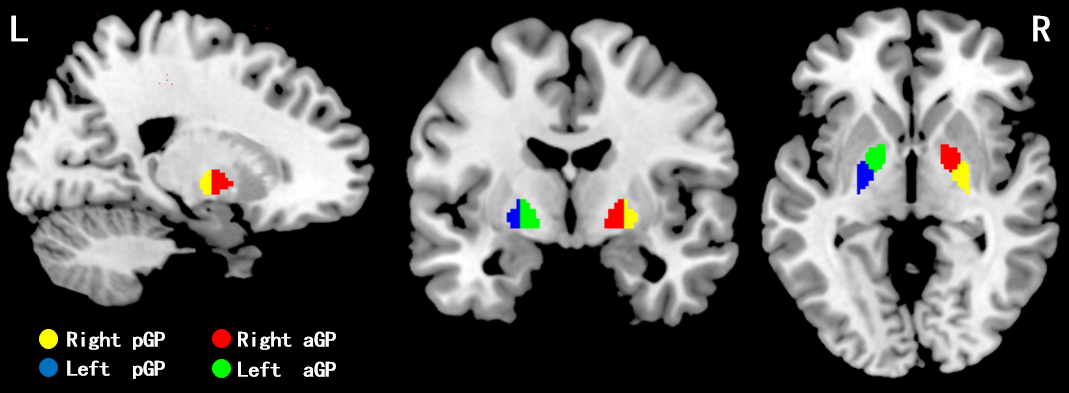


**Figure S1 Visualization of the subregions of globus pallidus. Four seeds of the globus pallidus in the bilateral hemisphere**

Abbreviations: aGP, anterior globus pallidus; pGP, posterior globus pallidus


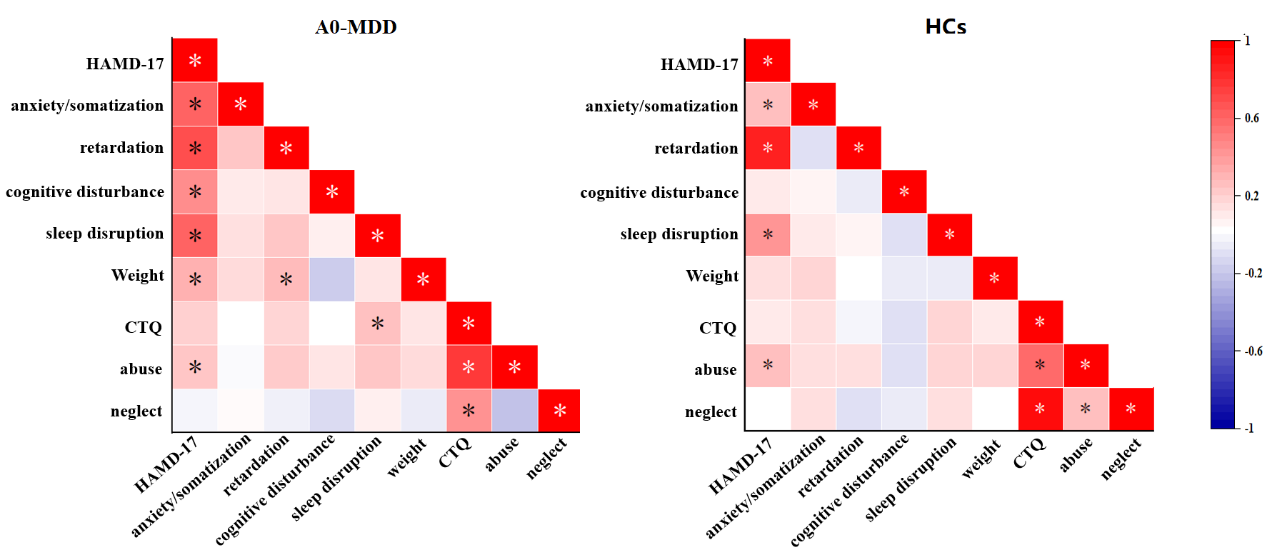


**Figure S2 Correlation analysis between depression severity and CM in AO-MDD and HCs group**Abbreviations: AO-MDD, adolescent-onset major depressive disorder; HCs, heathy controls; HAMD-17, 17-item Hamilton Depression Rating Scale; CTQ, Childhood Trauma Questionnaire, Hamilton Anxiety Scale.

**Table S1 Regions Showing Significant Differences in Functional Connectivity Among the AO-MDD and HCs group (n=155)**

| Region | MNI coordinates | | | Voxel size | T-value |
| --- | --- | --- | --- | --- | --- |
|  | X | Y | Z |  |  |
| Seed 1: left pGP | | | | | |
| Dorsolateral Prefrontal (BA8) | 28.5 | 11.5 | 53.5 | 304 | -4.77472 |

Abbreviations: AO-MDD, adolescent-onset major depressive disorder; HCs, heathy controls; pGP, posterior globus pallidus.

| **Table S2 Multiple linear regression of HAMA total by CTQ total and abuse and neglect with RSFC as a moderator** | | | | |
| --- | --- | --- | --- | --- |
| **Model 1: CTQ total** | **Estimate** | **SE** | **T** | **p** |
| Intercept | 14.0899 | 10.4560 | 1.3475 | 0.1827 |
| Age | 0.5049 | 0.8050 | 0.6271 | 0.5329 |
| Education | -0.4025 | 0.8430 | -0.4774 | 0.6347 |
| Female sex | -0.8310 | 2.1578 | -0.3851 | 0.7015 |
| CTQ total | 4.2173 | 2.9415 | 1.4337 | 0.1567 |
| RSFC | -94.9339 | 132.9651 | -0.7140 | 0.4779 |
| CTQ total X RSFC | 26.4435 | 59.0334 | 0.4479 | 0.6558 |
| **Model 2：abuse** | **Estimate** | **SE** | **T** | **p** |
| Intercept | 17.0555 | 8.4504 | 2.0183 | 0.0479 |
| Age | 0.4299 | 0.7692 | 0.5589 | 0.5783 |
| Education | -0.2314 | 0.8094 | -0.2859 | 0.7759 |
| Female sex | -2.1657 | 1.3275 | -1.6314 | 0.1079 |
| Abuse | 3.2329 | 1.6860 | 1.9175 | 0.0598 |
| RSFC | -13.7289 | 91.3732 | -0.1503 | 0.8811 |
| Abuse X RSFC | -9.1851 | 43.6936 | -0.2102 | 0.8342 |
| **Model 3：neglect** | **Estimate** | **SE** | **T** | **p** |
| Intercept | -0.0637 | 1.6924 | -0.0376 | 0.9701 |
| Age | -0.0180 | 0.2367 | -0.0760 | 0.9396 |
| Education | 0.0425 | 0.2303 | 0.1846 | 0.8540 |
| Female sex | 0.2019 | 0.3129 | 0.6454 | 0.5207 |
| Neglect | 0.3426 | 0.2447 | 1.4002 | 0.1657 |
| RSFC | 7.3107 | 5.3216 | 1.3738 | 0.1737 |
| Neglect X RSFC | -4.8112 | 3.1088 | -1.5476 | 0.1260 |

Abbreviations: HAMA, Hamilton Anxiety Rating Scale; RSFC, resting-state functional connectivity; CTQ, Childhood Trauma Questionnaire.

**Table S3 Regions Showing Significant Differences in Functional Connectivity Among the AO-MDD and HCs group in female participants (n=92)**

| Region | MNI coordinates | | | Voxel size(mm^2) | T-value |
| --- | --- | --- | --- | --- | --- |
|  | X | Y | Z |  |  |
| Seed 1: left pGP | | | | | |
| Dorsolateral Prefrontal (BA8) | 28.5 | 9.5 | 53.5 | 184 | -4.469 |

Abbreviations: AO-MDD, adolescent-onset major depressive disorder; HCs, heathy controls; pGP, posterior globus pallidus.
